# When the Heart Reveals Lupus: Severe Dilated Cardiomyopathy as the Initial Manifestation of Systemic Lupus Erythematosus

**DOI:** 10.7759/cureus.116094

**Published:** 2026-09-11

**Authors:** Mohamed Malki, Nadia Loudiyi, Hafsa Erregui, Meriem Elmarzouki, Najat Mouine

**Affiliations:** 1 Cardiology Department, Mohammed V Military Teaching Hospital, Rabat, MAR; 2 Cardiology B Department, Mohammed V University, Rabat, MAR

**Keywords:** cardiac magnetic resonance, dilated cardiomyopathy, heart failure, lupus myocarditis, multimodality imaging, systemic lupus erythematosus

## Abstract

Systemic lupus erythematosus (SLE) is a multisystem autoimmune disease with heterogeneous cardiovascular involvement, while clinically overt lupus myocarditis remains uncommon. We report the case of a 50-year-old woman with no previous cardiovascular or autoimmune disease who presented with acute decompensated heart failure after a two-year history of progressive exertional dyspnea. Transthoracic echocardiography revealed severe biventricular dilated cardiomyopathy with a left ventricular ejection fraction of 21% and profoundly reduced global longitudinal strain (-5.0%). Cardiac biomarkers were markedly elevated, whereas coronary angiography showed normal epicardial coronary arteries. Autoimmune testing revealed high-titer antinuclear antibodies, elevated anti-double-stranded DNA and anti-Smith antibodies, and complement consumption, despite the absence of typical cutaneous, articular, renal, or neurological manifestations of SLE. Cardiac magnetic resonance demonstrated subepicardial late gadolinium enhancement involving the anterolateral wall in a non-ischemic distribution. The integration of clinical, immunological, coronary, and multimodality imaging findings supported clinically suspected lupus myocarditis as the inaugural manifestation of previously unrecognized SLE. Guideline-directed heart failure therapy and immunosuppressive treatment were initiated, with early clinical improvement and a decrease in cardiac biomarkers. In the absence of endomyocardial biopsy, the diagnosis remained clinically and imaging-supported rather than histologically confirmed. This case highlights an unusual presentation of SLE revealed by severe biventricular dilated cardiomyopathy and emphasizes the pivotal role of multimodality imaging and targeted autoimmune testing in identifying an inflammatory etiology of otherwise unexplained non-ischemic cardiomyopathy.

## Introduction

Systemic lupus erythematosus (SLE) is a multisystem autoimmune disease with a broad spectrum of cardiovascular manifestations, including pericardial, valvular, coronary, and myocardial involvement [1]. Although subclinical myocardial abnormalities may be relatively frequent, clinically overt lupus myocarditis (LM) remains uncommon, affecting approximately 5-10% of patients, and may range from mild ventricular dysfunction to acute heart failure, dilated cardiomyopathy, and cardiogenic shock [1]. Lupus-related myocardial inflammation can cause cardiomyocyte injury, myocardial dysfunction, and adverse ventricular remodeling, which may ultimately result in a dilated cardiomyopathy phenotype [1]. Importantly, myocardial involvement may precede the recognition of SLE, making diagnosis particularly challenging when classical extracardiac manifestations are absent [2].

The diagnosis of LM is challenging because of the absence of a single specific diagnostic test and relies on the integration of clinical presentation, cardiac biomarkers, echocardiographic findings, immunological evidence of SLE, and cardiac magnetic resonance (CMR), together with the exclusion of alternative causes of myocardial injury. CMR plays a central role in suspected inflammatory myocardial involvement by combining ventricular functional assessment with myocardial tissue characterization and identification of non-ischemic patterns of myocardial injury, including late gadolinium enhancement (LGE) [3]. Echocardiographic global longitudinal strain (GLS), a measure of longitudinal myocardial deformation, may provide additional information regarding myocardial systolic dysfunction. However, CMR findings are not etiologically specific and must be interpreted within the clinical, biological, and immunological context [3,4].

We report an unusual case of a 50-year-old woman without previously known SLE or overt extracardiac manifestations who presented with severe decompensated heart failure revealing biventricular dilated cardiomyopathy with a left ventricular ejection fraction (LVEF) of 21%. Normal epicardial coronary arteries, a strongly supportive lupus immunological profile, and subepicardial anterolateral LGE on CMR supported clinically suspected lupus-related inflammatory myocardial involvement. This case is notable for severe biventricular dilated cardiomyopathy as the predominant inaugural manifestation of otherwise clinically silent SLE, highlighting the value of multimodality imaging and integrated clinical and immunological assessment in identifying an underlying inflammatory etiology.

## Case presentation

A 50-year-old woman with no known cardiovascular disease, modifiable cardiovascular risk factors, or significant past medical history presented to the emergency department with worsening dyspnea at rest over the preceding two days. Her symptoms had developed on a background of progressive exertional dyspnea evolving for approximately two years and corresponding to New York Heart Association (NYHA) functional class III before the current deterioration [5].

A detailed history was obtained to investigate potential cardiac and extracardiac causes of her chronic dyspnea and underlying cardiomyopathy. She denied chest pain, exertional angina, palpitations, syncope, or presyncope and had no history of hypertension, diabetes mellitus, dyslipidemia, smoking, or known coronary artery disease. There was no history of excessive alcohol consumption, recreational drug use, exposure to cardiotoxic agents, recent viral-like illness, thyroid disease, or other known metabolic disorders. Her family history was negative for cardiomyopathy, heart failure, or sudden cardiac death.

Given the possibility of an underlying systemic or autoimmune disorder, the history was specifically revisited for extracardiac manifestations. She denied unexplained fever, significant weight loss, photosensitivity, cutaneous rash, oral or nasal ulcerations, inflammatory arthralgia or arthritis, Raynaud phenomenon, sicca symptoms, alopecia, renal disease, or previous venous or arterial thrombosis. She had two previous full-term pregnancies without spontaneous abortion or recurrent pregnancy loss.

On admission, the patient was conscious, alert, and afebrile. Her blood pressure was 100/80 mmHg, heart rate was 100 beats/min with a regular rhythm, and peripheral oxygen saturation was 85% on room air. Cardiac auscultation revealed holosystolic murmurs consistent with mitral and tricuspid regurgitation, without a diastolic murmur or pericardial friction rub. Signs of systemic venous congestion included jugular venous distension, positive hepatojugular reflux, and bilateral pitting lower-limb edema extending to the knees. Peripheral pulses were palpable, and the extremities remained warm without clinical evidence of peripheral hypoperfusion.

Pulmonary examination revealed bilateral inspiratory crackles extending over the lower two-thirds of both lung fields, consistent with significant pulmonary congestion. Focused extracardiac examination showed no cutaneous lesions, oral or nasal ulcerations, alopecia, synovitis, joint swelling, or other apparent musculoskeletal abnormalities. Neurological examination was unremarkable. Abdominal examination revealed no clinically detectable hepatosplenomegaly or ascites, and no peripheral lymphadenopathy was identified.

Overall, the clinical presentation was consistent with severe biventricular congestive heart failure with resting hypoxemia and both pulmonary and systemic congestion, without signs of peripheral hypoperfusion. Importantly, despite the severity of the cardiac presentation, there were no overt extracardiac manifestations initially suggesting an underlying connective tissue disease.

Given the severity of the acute decompensation, the patient was admitted to the cardiac intensive care unit for close hemodynamic and respiratory monitoring. Initial management included oxygen therapy and intravenous loop diuretics, resulting in progressive improvement in respiratory distress and peripheral congestion.

The admission 12-lead electrocardiogram showed sinus rhythm at approximately 100 beats/min, with a PR interval of approximately 200 ms, consistent with borderline atrioventricular conduction delay. The QRS duration was approximately 120 ms with left bundle branch block morphology and associated secondary ST-T repolarization abnormalities. The corrected QT interval (QTc) was 342 ms (Figure 1).

**Figure 1:**
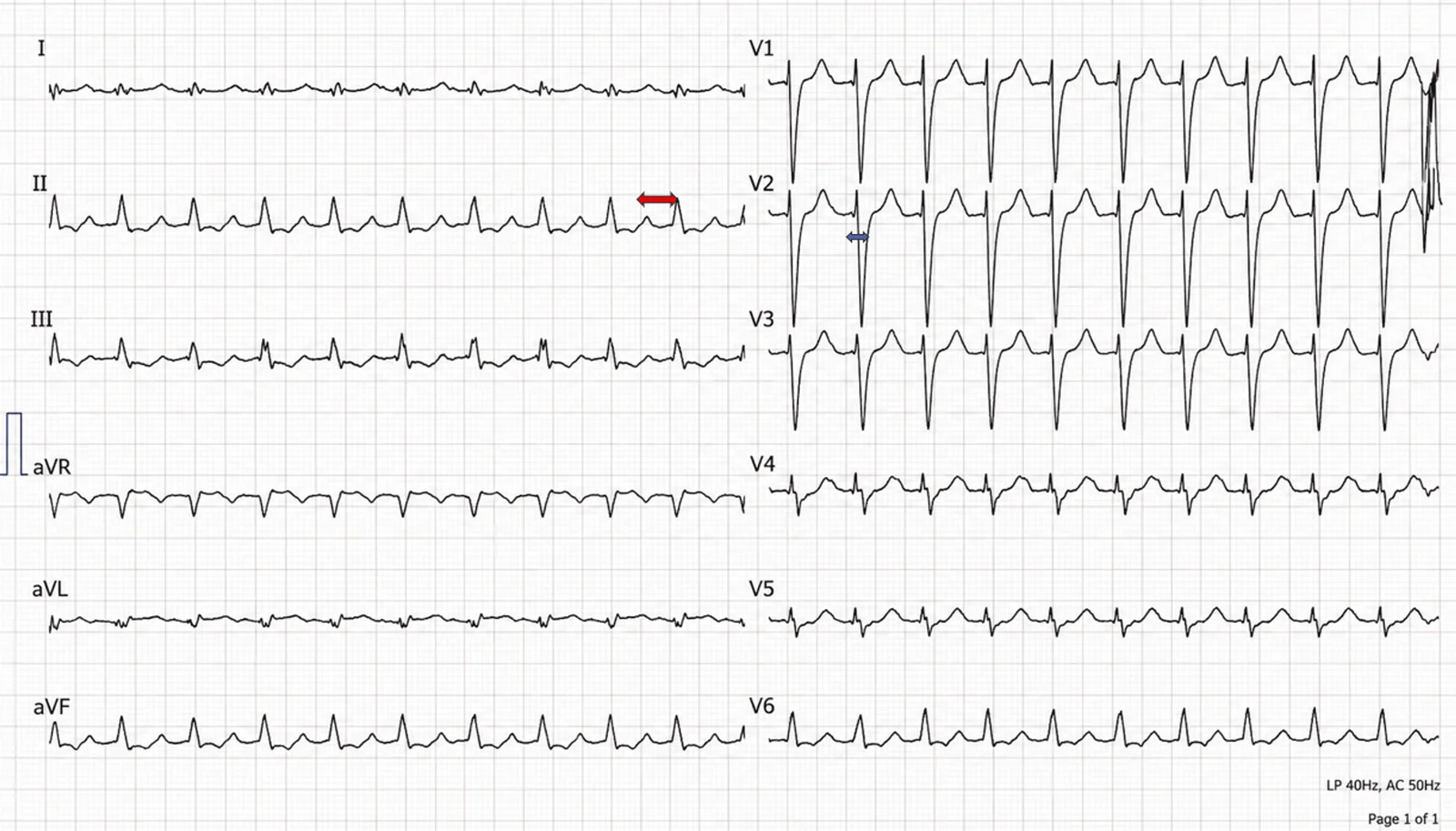
Admission 12-lead electrocardiogram showing borderline atrioventricular conduction delay and left bundle branch block. Admission 12-lead electrocardiogram showing sinus rhythm at approximately 100 beats/min with a borderline prolonged PR interval (≈200 ms; red arrow) and left bundle branch block (QRS duration ≈120 ms; blue arrow), associated with secondary ST–T repolarization abnormalities.

A chest radiograph demonstrated marked cardiomegaly with a downward-displaced cardiac apex extending below the level of the left hemidiaphragm, together with bilateral hilar vascular prominence consistent with pulmonary vascular congestion. No focal pulmonary parenchymal opacity was identified (Figure 2).

**Figure 2:**
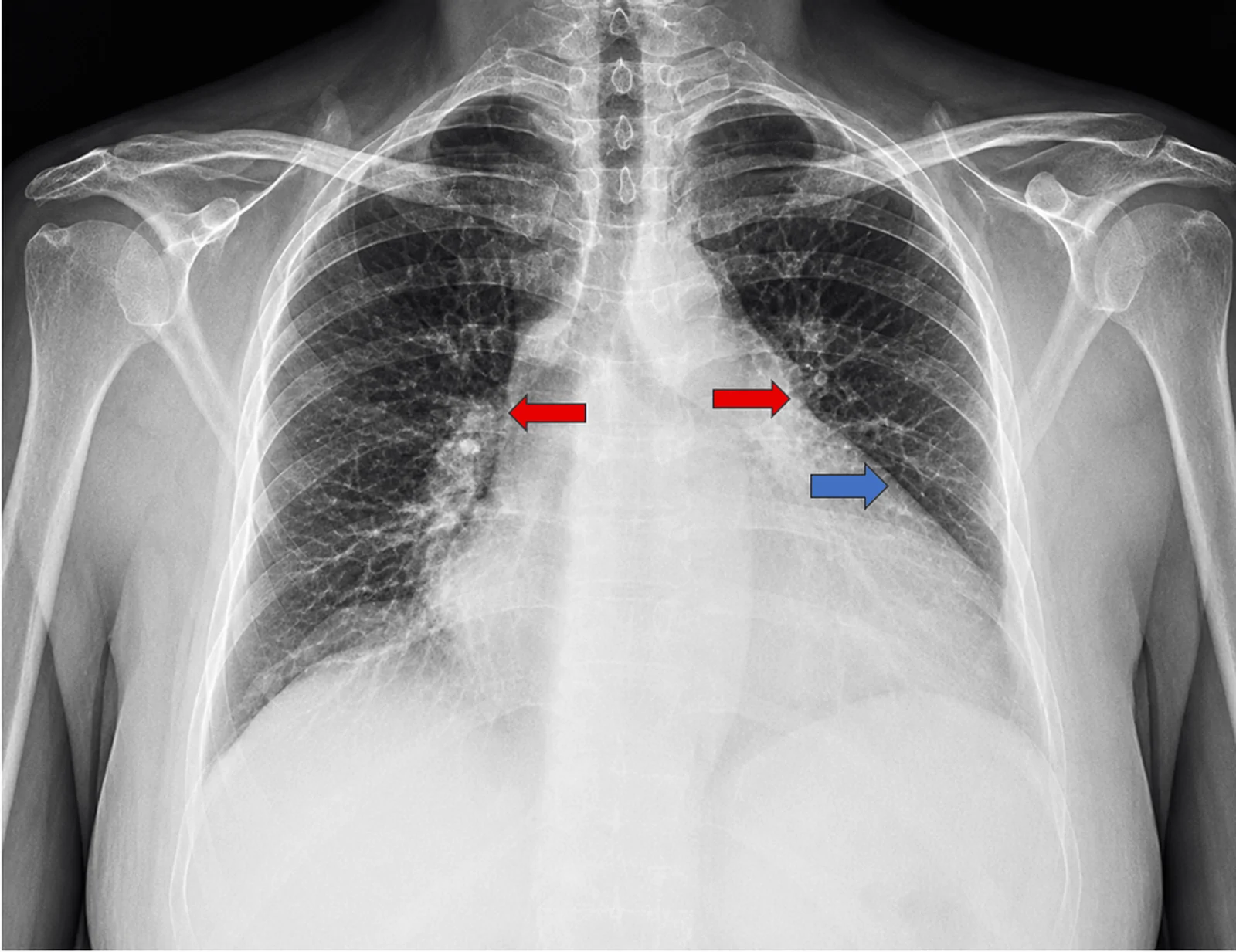
Chest radiograph showing cardiomegaly and pulmonary vascular congestion. Frontal chest radiograph demonstrating marked cardiomegaly with bilateral hilar vascular prominence consistent with pulmonary vascular congestion (red arrows). The blue arrow indicates straightening of the left middle cardiac border, with loss of the normal concavity of the pulmonary artery segment.

Transthoracic echocardiography (TTE) performed on admission revealed a markedly dilated left ventricle, with an end-diastolic diameter of 68 mm and an end-diastolic volume of 241 mL. Left ventricular systolic function was severely impaired, with a left ventricular ejection fraction (LVEF) of 21%. Diffuse severe hypokinesia with regional heterogeneity and mechanical dyssynchrony was observed. Speckle-tracking echocardiography demonstrated profound impairment of longitudinal deformation, with a global longitudinal strain (GLS) of -5.0%, predominantly involving the septal and anteroseptal segments. Paradoxical systolic deformation of several septal segments was observed in the setting of left bundle branch block.

The right ventricle was also dilated, with reduced longitudinal systolic function as indicated by a tricuspid annular systolic velocity (S′) of 9 cm/s, supporting biventricular involvement. Both atria were enlarged, with left and right atrial areas of 36.35 cm² and 22.19 cm², respectively. Mild functional mitral and tricuspid regurgitation were present. Peak tricuspid regurgitation velocity was 3.0 m/s, with an estimated systolic pulmonary artery pressure of approximately 51 mmHg, based on an estimated right atrial pressure of 15 mmHg. The inferior vena cava was dilated with reduced respiratory collapse. A minimal pericardial effusion was present without signs of hemodynamic compromise.

Overall, echocardiography demonstrated severe dilated cardiomyopathy with biventricular systolic dysfunction, profoundly impaired left ventricular longitudinal deformation, and elevated pulmonary pressures (Figure 3).

**Figure 3:**
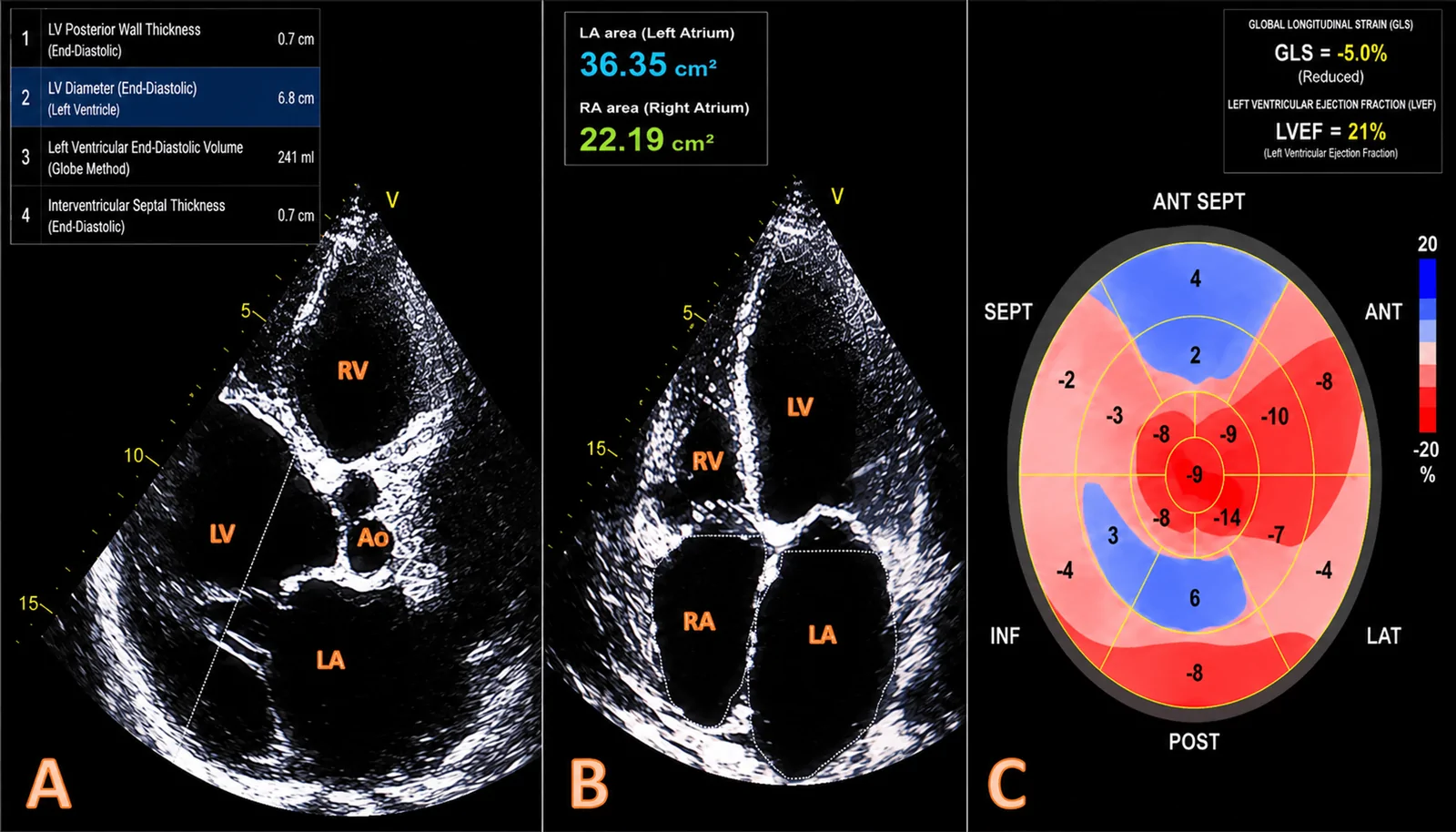
Transthoracic echocardiographic assessment at admission. (A) Parasternal long-axis view demonstrates marked left ventricular dilatation (left ventricular end-diastolic diameter, 68 mm; end-diastolic volume, 241 mL). (B) Apical four-chamber view shows biatrial enlargement (left atrial area, 36.35 cm²; right atrial area, 22.19 cm²) and severe left ventricular systolic dysfunction, with a left ventricular ejection fraction (LVEF) of 21%. (C) Speckle-tracking echocardiography demonstrates severely reduced global longitudinal strain (GLS, -5.0%), with marked regional heterogeneity and prominent septal mechanical dysfunction. Overall, these findings demonstrate severe dilated cardiomyopathy with profound left ventricular systolic and longitudinal functional impairment.

Initial laboratory investigations showed an unremarkable complete blood count, electrolyte profile, hepatic function, inflammatory markers, and coagulation profile (Table 1). Renal function was preserved, with a serum creatinine level of 74 µmol/L and an estimated glomerular filtration rate of 92 mL/min/1.73 m². C-reactive protein and procalcitonin levels were within normal limits. Coagulation parameters were also normal, with a prothrombin time of 92% and an international normalized ratio (INR) of 1.05. In contrast, cardiac biomarkers were markedly elevated, with a high-sensitivity troponin level of 300 ng/L and an N-terminal pro-B-type natriuretic peptide (NT-proBNP) level of 30,000 pg/mL, consistent with myocardial injury and severe hemodynamic cardiac stress.

**Table 1:** Laboratory findings on admission. Complete blood count, electrolyte levels, renal and hepatic function, inflammatory markers, and coagulation parameters were within reference ranges, whereas high-sensitivity cardiac troponin (hs-troponin) and N-terminal pro-B-type natriuretic peptide (NT-proBNP) levels were markedly elevated. ALT, alanine aminotransferase; AST, aspartate aminotransferase; CRP, C-reactive protein; eGFR, estimated glomerular filtration rate; INR, international normalized ratio; NT-proBNP, N-terminal pro-B-type natriuretic peptide; PT, prothrombin time; hs-troponin, high-sensitivity cardiac troponin.

Laboratory parameter	Patient value	Reference range
Hemoglobin	13.1 g/dL	12.0–16.0 g/dL
White blood cell count	7,800/mm³	4,000–10,000/mm³
Neutrophils	4,900/mm³	1,500–7,500/mm³
Lymphocytes	2,200/mm³	1,000–4,000/mm³
Platelet count	238,000/mm³	150,000–400,000/mm³
Sodium	138 mmol/L	135–145 mmol/L
Potassium	4.2 mmol/L	3.5–5.0 mmol/L
Chloride	103 mmol/L	98–107 mmol/L
Urea	0.32 g/L	0.15–0.45 g/L
Creatinine	74 µmol/L	45–90 µmol/L
eGFR (CKD-EPI)	92 mL/min/1.73 m²	≥90 mL/min/1.73 m²
AST	24 U/L	<35 U/L
ALT	21 U/L	<35 U/L
CRP	3.2 mg/L	<5 mg/L
Procalcitonin	0.04 ng/mL	<0.10 ng/mL
Prothrombin time (PT)	92%	70–100%
INR	1.05	0.8–1.2
hs-Troponin	300 ng/L	<14 ng/L
NT-proBNP	30,000 pg/mL	<300 pg/mL

Given the severe left ventricular systolic dysfunction, regional wall-motion abnormalities, and elevated cardiac troponin levels, an ischemic etiology was considered despite the absence of anginal symptoms or conventional cardiovascular risk factors. Coronary angiography demonstrated normal epicardial coronary arteries without significant stenosis, supporting a non-ischemic etiology of the cardiomyopathy (Figure 4).

**Figure 4:**
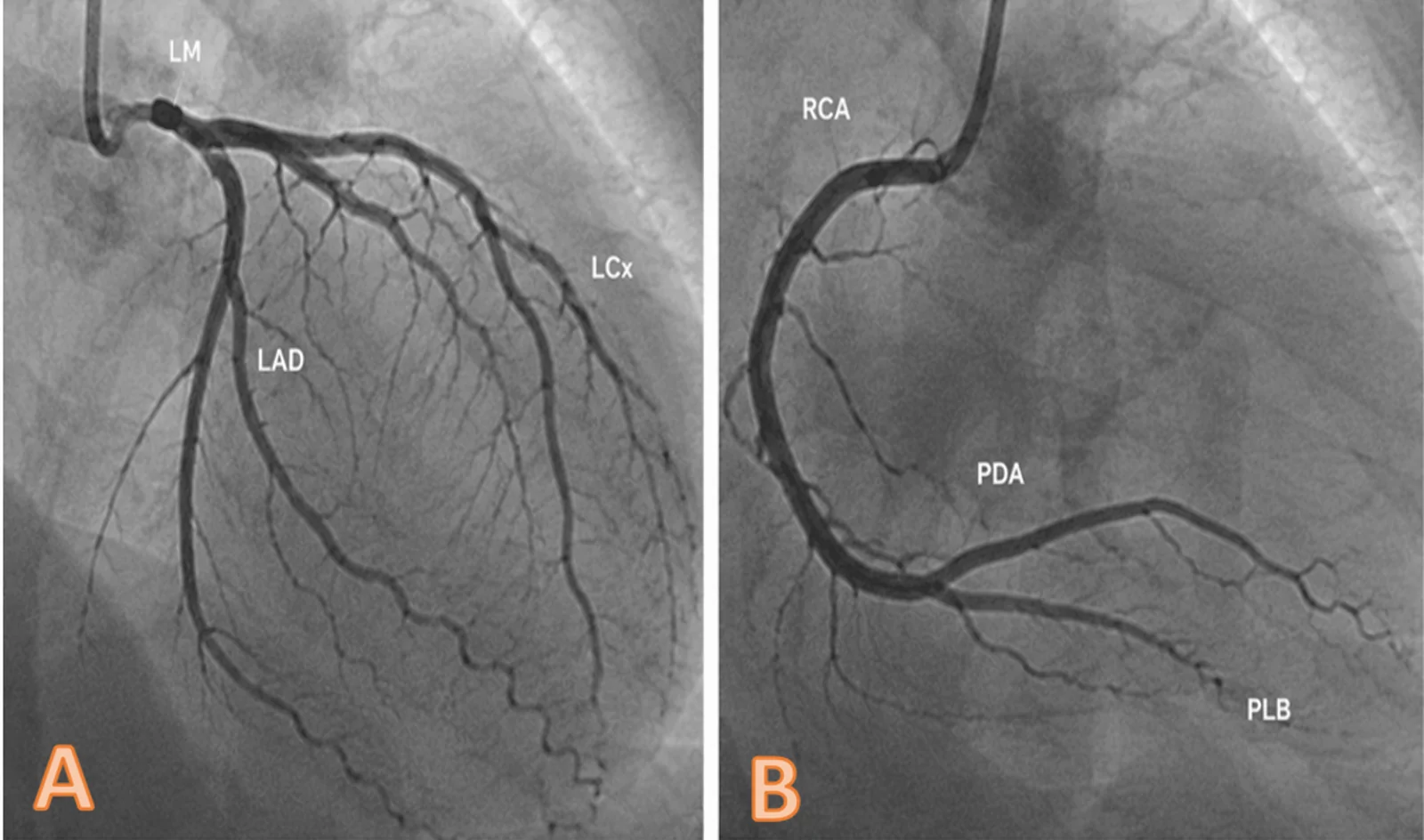
Coronary angiography showing normal epicardial coronary arteries without significant obstructive lesions. (A) Left coronary angiography demonstrating a patent left main coronary artery (LM), left anterior descending artery (LAD), and left circumflex artery (LCx). (B) Right coronary angiography demonstrating a patent right coronary artery (RCA), posterior descending artery (PDA), and posterolateral branch (PLB).

Following exclusion of obstructive coronary artery disease, an extensive etiological work-up for non-ischemic dilated cardiomyopathy was undertaken. Autoimmune testing revealed strongly positive antinuclear antibodies (ANA) at a titer of 1:1280 with a homogeneous pattern, markedly elevated anti-double-stranded DNA (anti-dsDNA) antibodies (185 IU/mL), and positive anti-Smith (anti-Sm) antibodies (62 U/mL). Complement levels were reduced, with C3 at 0.55 g/L and C4 at 0.07 g/L, indicating complement consumption. The antiphospholipid profile, including lupus anticoagulant, anticardiolipin IgG/IgM antibodies, and anti-β2-glycoprotein I IgG/IgM antibodies, was negative (Table 2).

**Table 2:** Autoimmune and antiphospholipid work-up. Autoimmune and antiphospholipid testing revealed a serological profile strongly supportive of systemic lupus erythematosus, characterized by high-titer antinuclear antibodies (ANA), elevated anti-double-stranded DNA (anti-dsDNA) and anti-Smith (anti-Sm) antibodies, and complement consumption, with a negative antiphospholipid antibody profile. ANA, antinuclear antibodies; ANCA, antineutrophil cytoplasmic antibodies; anti-β2GPI, anti-beta-2 glycoprotein I antibodies; anti-CCP, anti-cyclic citrullinated peptide antibodies; anti-dsDNA, anti-double-stranded DNA antibodies; anti-La/SSB, anti-La/Sjögren’s-syndrome-related antigen B antibodies; anti-RNP, anti-ribonucleoprotein antibodies; anti-Ro/SSA, anti-Ro/Sjögren’s-syndrome-related antigen A antibodies; anti-Sm, anti-Smith antibodies; C3, complement component 3; C4, complement component 4; IgG, immunoglobulin G; IgM, immunoglobulin M; MPO, myeloperoxidase; PR3, proteinase 3.

Investigation	Result	Reference range
Autoimmune screening		
ANA	Positive, 1:1280	<1:80
ANA pattern	Homogeneous	—
Anti-dsDNA	185 IU/mL	<30 IU/mL
Anti-Sm	62 U/mL	<10 U/mL
Anti-RNP	38 U/mL	<10 U/mL
Anti-Ro/SSA	Negative	Negative
Anti-La/SSB	Negative	Negative
Rheumatoid factor	Negative	<14 IU/mL
Anti-CCP	Negative	<20 U/mL
PR3-ANCA	Negative	Negative
MPO-ANCA	Negative	Negative
Complement C3	0.55 g/L	0.90–1.80 g/L
Complement C4	0.07 g/L	0.10–0.40 g/L
Antiphospholipid profile		
Lupus anticoagulant	Negative	Negative
Anticardiolipin IgG	4 GPL U/mL	<20 GPL U/mL
Anticardiolipin IgM	3 MPL U/mL	<20 MPL U/mL
Anti-β2-glycoprotein I IgG	5 U/mL	<20 U/mL
Anti-β2-glycoprotein I IgM	4 U/mL	<20 U/mL

According to the 2019 European Alliance of Associations for Rheumatology/American College of Rheumatology (EULAR/ACR) classification criteria, the positive ANA fulfilled the mandatory entry criterion [6]. The pericardial effusion was counted as a serosal clinical criterion (5 points). In addition, the presence of SLE-specific antibodies (anti-dsDNA and anti-Sm; 6 points within the same immunological domain) and low C3 and C4 levels (4 points) yielded a total classification score of 15 points, exceeding the ≥10-point threshold required for SLE classification [6].

The remainder of the etiological work-up did not identify an alternative cause of the cardiomyopathy. Infectious screening, including HIV, hepatitis B, hepatitis C, and syphilis serologies, was negative. Endocrine and metabolic investigations were unremarkable, including normal thyroid function (TSH, 1.84 mIU/L; free T4, 14.8 pmol/L), glycemic status, calcium, magnesium, and ferritin levels. Serum protein electrophoresis revealed polyclonal hypergammaglobulinemia without a monoclonal component, while tumor markers were within normal limits (Table 3).

**Table 3:** Infectious, endocrine, metabolic, and neoplastic investigations performed as part of the etiological work-up for non-ischemic dilated cardiomyopathy. Comprehensive etiological work-up for non-ischemic dilated cardiomyopathy, including infectious, endocrine, metabolic, serum protein electrophoresis, and neoplastic investigations, showing no alternative underlying etiology. HIV, human immunodeficiency virus; HBsAg, hepatitis B surface antigen; anti-HBc, hepatitis B core antibody; HCV, hepatitis C virus; TPHA, Treponema pallidum hemagglutination assay; VDRL, Venereal Disease Research Laboratory; TSH, thyroid-stimulating hormone; FT4, free thyroxine; HbA1c, glycated hemoglobin; SPEP, serum protein electrophoresis; CEA, carcinoembryonic antigen; CA 19-9, carbohydrate antigen 19-9; CA-125, cancer antigen 125; AFP, alpha-fetoprotein.

Investigation	Result	Reference range
Infectious screening		
HIV 1/2 serology	Negative	Negative
HBsAg / anti-HBc	Negative	Negative
Anti-HCV antibodies	Negative	Negative
Syphilis serology (TPHA/VDRL)	Negative	Negative
Endocrine and metabolic work-up		
TSH	1.84 mIU/L	0.4–4.0 mIU/L
Free T4	14.8 pmol/L	12–22 pmol/L
Fasting glucose	0.91 g/L	0.70–1.00 g/L
HbA1c	5.4%	<5.7%
Calcium	2.32 mmol/L	2.20–2.60 mmol/L
Magnesium	0.84 mmol/L	0.70–1.00 mmol/L
Ferritin	86 ng/mL	15–150 ng/mL
Serum protein electrophoresis		
SPEP	Polyclonal hypergammaglobulinemia	No monoclonal component
Tumor markers		
CEA	2.1 ng/mL	<5 ng/mL
CA 19-9	14 U/mL	<37 U/mL
CA-125	18 U/mL	<35 U/mL
AFP	3.2 ng/mL	<10 ng/mL

Cardiac magnetic resonance (CMR) imaging was subsequently performed for further morphological and tissue characterization. Cine sequences confirmed a dilated cardiomyopathy phenotype, with marked left ventricular dilatation and severe global systolic dysfunction (left ventricular ejection fraction (LVEF), 21%). The right ventricle was also dilated, with mildly impaired systolic function (right ventricular ejection fraction (RVEF), 49%). A minimal circumferential pericardial effusion was also noted (Figure 5).

**Figure 5:**
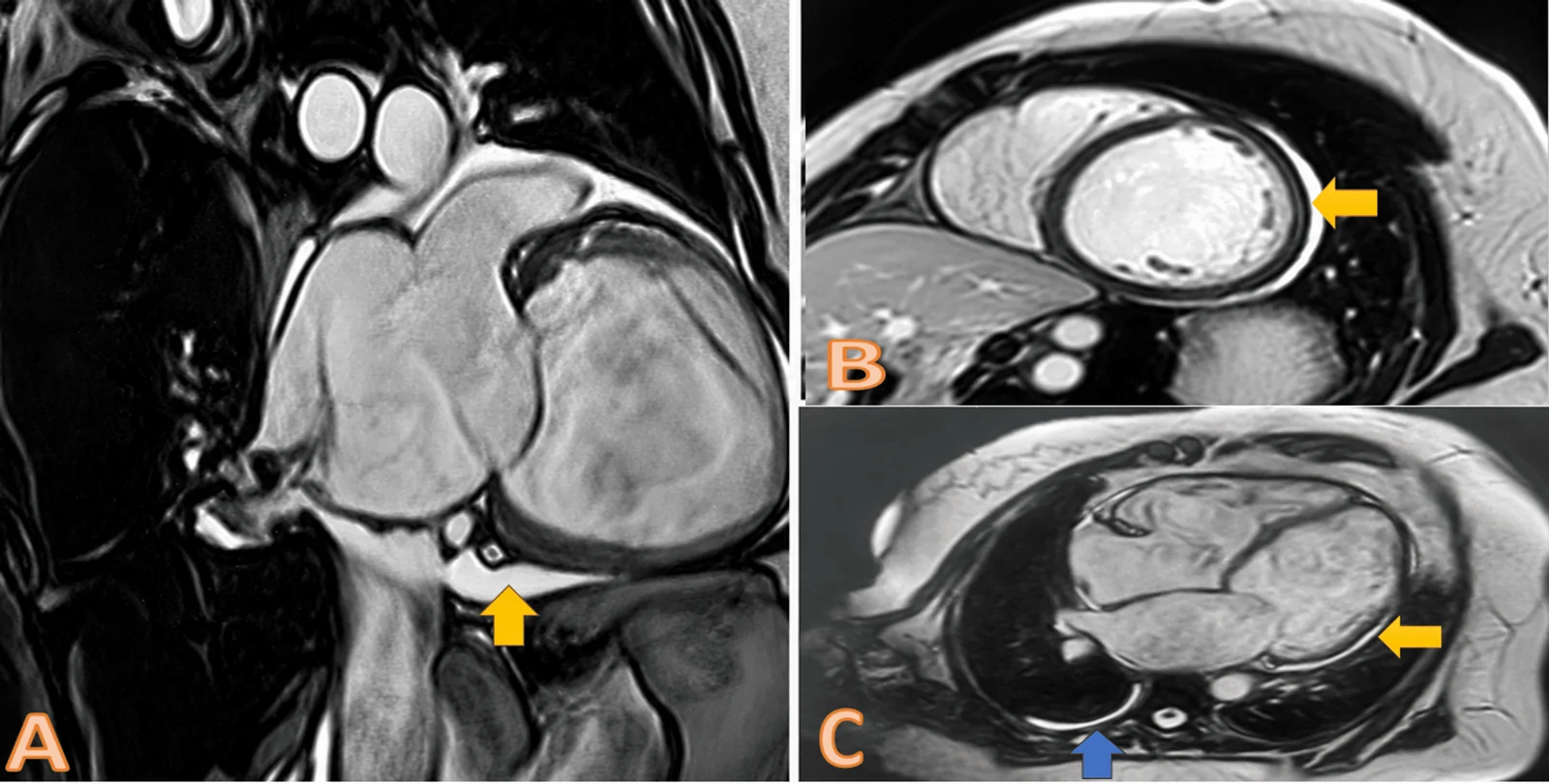
Cardiac magnetic resonance cine imaging demonstrating a dilated cardiomyopathy phenotype. (A) Two-chamber, (B) short-axis, and (C) four-chamber cine views demonstrate marked left ventricular dilatation with severe global systolic dysfunction. A small circumferential pericardial effusion is visible (yellow arrows), while a small right pleural effusion is seen on the four-chamber view (blue arrow).

Late gadolinium enhancement (LGE) imaging demonstrated subepicardial enhancement of the anterolateral wall in a non-ischemic distribution (Figure 6). Quantitative T1 and T2 mapping data were not available; therefore, the updated Lake Louise Criteria for acute myocardial inflammation could not be fully assessed. Nevertheless, in the context of myocardial injury reflected by elevated cardiac troponin levels, normal epicardial coronary arteries, and a strongly supportive SLE immunological profile, the CMR findings supported clinically suspected inflammatory myocardial involvement attributable to SLE. In the absence of endomyocardial biopsy, the diagnosis was considered clinically suspected lupus myocarditis rather than histologically confirmed myocarditis.

**Figure 6:**
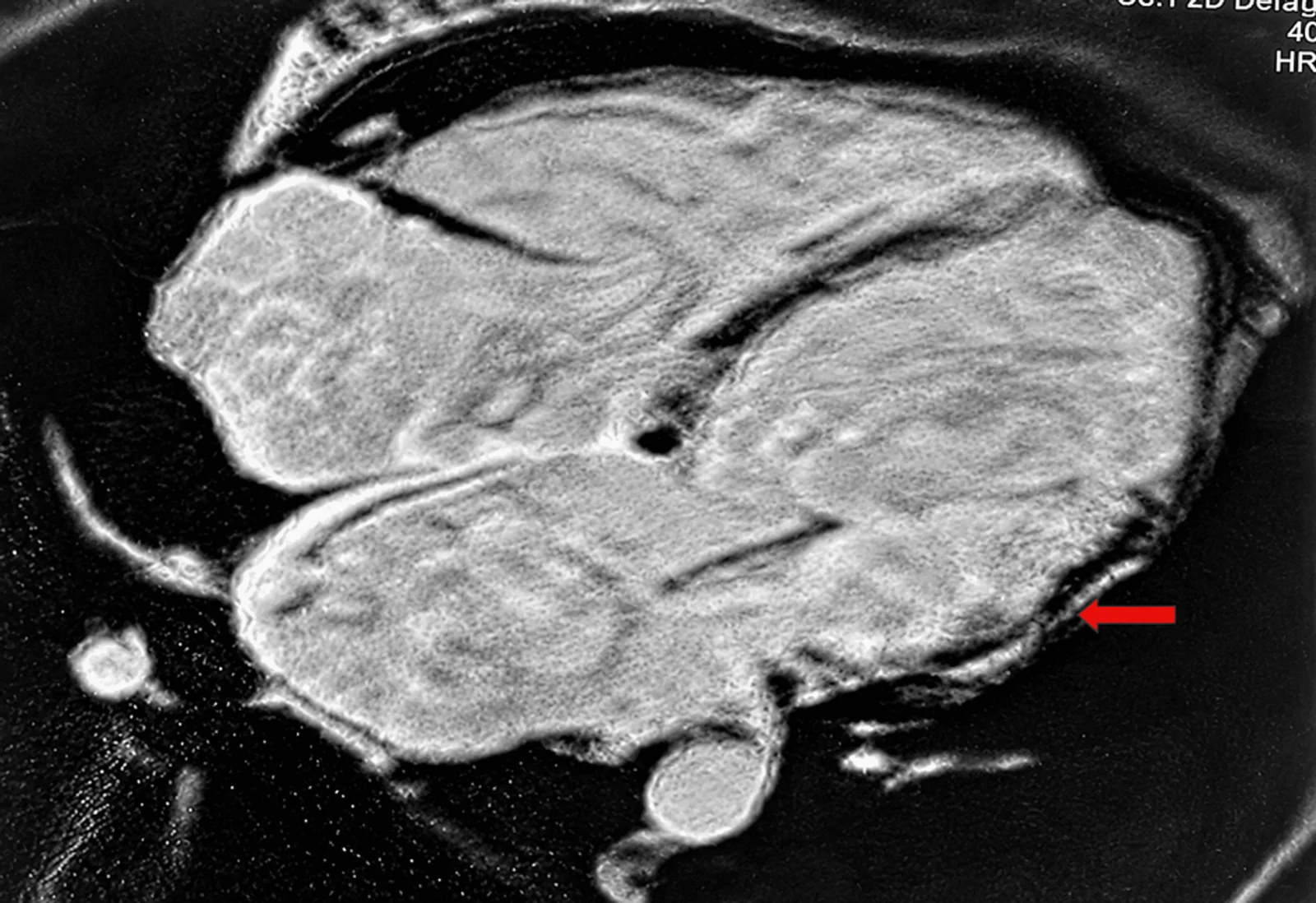
Cardiac magnetic resonance late gadolinium enhancement (LGE) imaging. Four-chamber late gadolinium enhancement (LGE) image demonstrates subepicardial enhancement involving the anterolateral left ventricular wall (red arrow), consistent with a non-ischemic pattern of myocardial injury. In the appropriate clinical and immunological context, this finding supports clinically suspected lupus myocarditis.

The overall findings were reviewed by a multidisciplinary team involving cardiologists, cardiac imaging specialists, electrophysiologists, and internal medicine/rheumatology specialists. The combination of severe non-ischemic dilated cardiomyopathy with biventricular systolic dysfunction, elevated cardiac biomarkers, normal epicardial coronary arteries, a strongly supportive SLE immunological profile with complement consumption, and a non-ischemic subepicardial anterolateral late gadolinium enhancement (LGE) pattern on CMR supported lupus-related inflammatory myocardial involvement. The associated small pericardial effusion provided additional evidence of cardiac involvement. In the absence of endomyocardial biopsy, the diagnosis was considered clinically suspected lupus myocarditis rather than histologically confirmed myocarditis.

During the acute phase, the patient was managed in the cardiac intensive care unit with oxygen therapy and intravenous loop diuretics, resulting in progressive clinical decongestion and improvement in respiratory status. Once hemodynamically stabilized, guideline-directed medical therapy for heart failure with reduced ejection fraction was progressively introduced and titrated according to blood pressure, renal function, and serum potassium levels. This included sacubitril/valsartan 49/51 mg twice daily, bisoprolol 10 mg once daily, spironolactone 50 mg once daily, and dapagliflozin 10 mg once daily.

Given the severity of the suspected lupus-related inflammatory myocardial involvement and profound biventricular systolic dysfunction, intravenous methylprednisolone pulse therapy at 500 mg/day for three consecutive days was initiated after multidisciplinary discussion, followed by oral prednisone at 1 mg/kg/day with a planned progressive taper according to the clinical, biological, and cardiac response. Additional immunosuppressive therapy was considered in collaboration with the internal medicine/rheumatology team according to disease severity and subsequent clinical evolution.

Given the severely reduced LVEF (21%), left bundle branch block morphology, and marked mechanical dyssynchrony, arrhythmic risk and indications for device therapy were also evaluated. Twenty-four-hour Holter monitoring showed persistent sinus rhythm without episodes of non-sustained or sustained ventricular tachycardia. Immediate primary-prevention implantable cardioverter-defibrillator (ICD) implantation was deferred pending reassessment of ventricular function after optimization of guideline-directed medical and immunosuppressive therapy. Similarly, immediate cardiac resynchronization therapy (CRT) was not pursued because the QRS duration was approximately 120 ms. The multidisciplinary team therefore favored optimization of medical and immunosuppressive therapy followed by reassessment of LVEF, QRS duration, symptoms, ventricular remodeling, and arrhythmic risk after approximately three months. CRT and/or primary-prevention ICD therapy would subsequently be reconsidered if severe left ventricular systolic dysfunction and appropriate guideline-based indications persisted despite optimized therapy.

During early follow-up, the patient showed clinical improvement accompanied by a decrease in cardiac biomarkers. High-sensitivity troponin decreased from 300 ng/L at admission to 100 ng/L, while NT-proBNP decreased from 30,000 pg/mL to 20,000 pg/mL. At the time of manuscript preparation, the patient remained under active multidisciplinary follow-up, with guideline-directed heart failure therapy and immunosuppressive treatment being progressively optimized according to clinical and biological tolerance. Serial clinical, biomarker, electrocardiographic, and echocardiographic assessments were planned to evaluate the subsequent cardiac course. Repeat CMR was also considered to assess the evolution of myocardial abnormalities and residual myocardial injury. Longer-term clinical and imaging outcomes, including reassessment of LVEF, were not yet available.

## Discussion

Lupus myocarditis (LM) is an uncommon but potentially severe manifestation of systemic lupus erythematosus (SLE), clinically affecting approximately 5-10% of patients and ranging from subclinical myocardial injury to acute heart failure, dilated cardiomyopathy (DCM), arrhythmias, and cardiogenic shock [1]. Importantly, myocardial involvement may precede the recognition of SLE; in the multicenter series by Thomas et al., myocarditis was the inaugural manifestation in 17 of 29 patients (58.6%) [2]. Our case is particularly remarkable because severe biventricular DCM with a left ventricular ejection fraction (LVEF) of 21% revealed previously unrecognized SLE in a patient without typical cutaneous, articular, renal, or neurological manifestations.

The diagnosis of LM remains challenging because no single clinical, biological, or imaging finding is pathognomonic [1]. Cardiac magnetic resonance (CMR) plays a central role in suspected myocarditis by combining ventricular functional assessment with myocardial tissue characterization [3]. Non-ischemic late gadolinium enhancement (LGE), particularly involving the subepicardial or mid-wall layers, supports a non-ischemic pattern of myocardial injury [3,4]. In our patient, severe biventricular dysfunction, markedly reduced global longitudinal strain (GLS, -5.0%), elevated cardiac troponin levels, normal epicardial coronary arteries, and subepicardial anterolateral LGE supported inflammatory myocardial involvement rather than ischemic cardiomyopathy. The associated small pericardial effusion provided additional evidence of cardiac involvement. Quantitative T1 and T2 mapping data were not available; therefore, the updated Lake Louise Criteria for acute myocardial inflammation could not be fully assessed [3].

Several differential diagnoses were considered. Ischemic cardiomyopathy was excluded by normal coronary angiography and the non-ischemic LGE distribution [3]. Viral myocarditis represented an important differential diagnosis because CMR findings may substantially overlap with those of autoimmune myocarditis [4]. CMR can identify patterns suggestive of myocardial inflammation or injury but cannot independently establish their etiology [3,4]. In our patient, the absence of a preceding viral syndrome, negative infectious evaluation, high-titer antinuclear antibodies (ANA), elevated anti-double-stranded DNA (anti-dsDNA) and anti-Smith (anti-Sm) antibodies, and low C3 and C4 levels strongly favored lupus-related myocardial involvement. Toxic, metabolic, and endocrine causes were also considered unlikely after an extensive negative etiological work-up.

According to the 2019 European Alliance of Associations for Rheumatology/American College of Rheumatology (EULAR/ACR) classification criteria, ANA positivity fulfilled the mandatory entry criterion. The pericardial effusion was counted as a serosal clinical criterion (5 points) [6]. SLE-specific antibodies (anti-dsDNA and anti-Sm) contributed 6 points within the same immunological domain, while low C3 and C4 levels contributed 4 points, yielding a total classification score of 15 points and exceeding the ≥10-point threshold required for SLE classification [6]. This is particularly relevant in our patient, in whom cardiac involvement, including pericardial involvement, contributed to the initial recognition of SLE in the absence of other overt extracardiac manifestations.

Endomyocardial biopsy remains an important diagnostic tool in selected cases of suspected myocarditis, particularly when histological characterization is expected to influence management; however, its diagnostic yield may be limited by the focal distribution of myocardial inflammation [1,3]. Integration of clinical presentation, cardiac biomarkers, autoimmune serology, coronary assessment, and CMR can therefore provide strong supportive evidence when biopsy is not performed [1,3]. Accordingly, in the absence of endomyocardial biopsy, our case is best described as clinically suspected lupus myocarditis supported by clinical, immunological, and imaging findings rather than biopsy-proven myocarditis.

Treatment requires simultaneous management of heart failure and the underlying inflammatory process [1,7]. Guideline-directed medical therapy should be introduced and optimized in patients with reduced LVEF [8], while glucocorticoids remain a commonly used immunosuppressive treatment for clinically significant LM [1,7]. In severe presentations, high-dose intravenous corticosteroid pulses are frequently administered before transition to oral glucocorticoid therapy [1,7]. In our patient, intravenous methylprednisolone at 500 mg/day for three consecutive days was followed by oral prednisone at 1 mg/kg/day, with progressive tapering planned according to clinical, biological, and cardiac response. Additional immunosuppressive agents, including cyclophosphamide or mycophenolate mofetil, may be considered in severe organ involvement [7], although therapeutic strategies for LM remain largely supported by observational evidence. Improvement in cardiac function following intravenous cyclophosphamide has also been reported in severe lupus myocarditis [9].

Device therapy also requires careful consideration in patients presenting with severe left ventricular dysfunction. Despite an LVEF of 21%, primary-prevention implantable cardioverter-defibrillator (ICD) implantation was deferred during the initial phase to allow optimization of heart failure and immunosuppressive therapy followed by reassessment of ventricular function [8]. Twenty-four-hour Holter monitoring showed persistent sinus rhythm without episodes of non-sustained or sustained ventricular tachycardia. Although left bundle branch block morphology and marked mechanical dyssynchrony were present, immediate cardiac resynchronization therapy (CRT) was not pursued because the QRS duration was approximately 120 ms [8]. LVEF, electrical dyssynchrony, symptoms, and arrhythmic risk should therefore be reassessed after optimized therapy, with ICD and/or CRT reconsidered if guideline-based indications persist [8].

Early follow-up in our patient showed clinical improvement accompanied by a decrease in cardiac biomarkers. High-sensitivity cardiac troponin decreased from 300 ng/L at admission to 100 ng/L, while N-terminal pro-B-type natriuretic peptide (NT-proBNP) decreased from 30,000 pg/mL to 20,000 pg/mL. These findings indicate an early favorable clinical and biological response but do not establish recovery of ventricular function. Longer-term echocardiographic and CMR follow-up is therefore required before conclusions regarding ventricular recovery or reversibility can be drawn.

The prognosis of LM is heterogeneous. In the multicenter series by Thomas et al., 43% of evaluated patients recovered an LVEF ≥55% within one month and 81% had an LVEF ≥55% at the end of follow-up [2]. Nevertheless, severe presentations may be associated with cardiogenic shock, arrhythmias, conduction abnormalities, and persistent ventricular dysfunction [1,2]. Serial echocardiography is therefore important for assessing ventricular function and remodeling, while follow-up CMR may provide complementary information regarding the evolution of myocardial abnormalities and residual myocardial injury [1,3]. In our patient, long-term prognosis cannot yet be determined because follow-up imaging documenting the evolution of ventricular function is not yet available.

Overall, this case emphasizes that clinically suspected LM should be considered in otherwise unexplained non-ischemic DCM, even in the absence of classical extracardiac manifestations of SLE. Integration of multimodality imaging, coronary assessment, cardiac biomarkers, and targeted autoimmune testing can identify an underlying inflammatory and autoimmune context [1,3]. However, in the absence of endomyocardial biopsy and documented longitudinal recovery of ventricular function, diagnostic and prognostic conclusions should remain appropriately cautious. Continued clinical, biological, echocardiographic, and CMR follow-up is essential to assess treatment response, ventricular evolution, and subsequent indications for device therapy [3,8].

## Conclusions

This case highlights a rare and diagnostically challenging presentation of systemic lupus erythematosus (SLE), revealed by severe biventricular dilated cardiomyopathy as the predominant inaugural manifestation in the absence of overt extracardiac features. The combination of profound ventricular dysfunction, markedly impaired myocardial deformation, elevated cardiac biomarkers, normal epicardial coronary arteries, subepicardial anterolateral late gadolinium enhancement (LGE) on cardiac magnetic resonance (CMR), and a strongly supportive SLE immunological profile supported clinically suspected lupus-related inflammatory myocardial involvement. In the absence of endomyocardial biopsy, the diagnosis of lupus myocarditis remained clinically and imaging-supported rather than histologically confirmed. Lupus myocarditis should therefore be considered in the differential diagnosis of otherwise unexplained non-ischemic dilated cardiomyopathy, even when classical systemic manifestations of SLE are absent. Early recognition may influence immunosuppressive and heart failure management and guide subsequent reassessment of ventricular function and indications for device therapy.
